# Vestibular Dysfunction as a Novel Presentation of Middle Ear Neuroendocrine Tumor

**DOI:** 10.1055/a-2823-9080

**Published:** 2026-03-13

**Authors:** Amor Niksic, Rance JT Fujiwara, Brandon Isaacson

**Affiliations:** 1 Department of Otolaryngology – Head and Neck Surgery, University of Texas Southwestern Medical Center, Dallas, Texas, United States

**Keywords:** middle ear neuroendocrine tumor, cochlear–vestibular dysfunction, DOTA-TATE PET/MRI

## Abstract

**Objectives:**

This study aimed to contribute to the sparse literature on middle ear neuroendocrine tumors (MeNETs) by detailing a unique case of MeNET-associated cochlear–vestibular dysfunction and outlining diagnostic, therapeutic, and follow-up considerations.

**Design:**

A single-patient case report.

**Setting:**

A tertiary academic referral center.

**Participant:**

A 40-year-old male presenting with profound left sensorineural hearing loss (SNHL), left vestibular hypofunction, and a middle ear mass.

**Main Outcome Measures:**

Histopathologic confirmation of MeNET, radiological identification of residual disease, postoperative symptom progression, and rehabilitation outcome.

**Results:**

Clinical evaluation, imaging, and histopathology confirmed the diagnosis of MeNET. Immunohistochemistry revealed positivity for chromogranin, synaptophysin, insulinoma-associated protein 1 (INSM1), cytokeratin mouse monoclonal antibody (CAM 5.2). An endoscopic middle ear exploration with tympanoplasty and canalplasty was performed, followed by vestibular rehabilitation. Surveillance with DOTA-Tyr3-octreotate PET/MRI identified residual disease, leading to a revision mastoidectomy. The patient experienced persistent imbalance postoperatively, requiring vestibular rehabilitation, and profound SNHL, for which a hearing aid evaluation was recommended.

**Conclusions:**

This case represents the first report of MeNET-associated cochlear–vestibular dysfunction. The significance of this report lies in the unique clinical presentation, the role of advanced imaging for surveillance, and the need for revision mastoidectomy following an initial endoscopic approach. This report discusses the challenges in achieving complete tumor clearance and emphasizes the need for continued case documentation and research into adjunctive therapies.

## Introduction

Neuroendocrine tumors of the middle ear (MeNETs) represent a unique subset of neuroendocrine neoplasms. These tumors pose diagnostic challenges due to the complex middle ear anatomy and limited reported cases. 1 Their rarity and non-specific symptoms often lead to frequent misdiagnoses and delayed diagnosis. 2

In this report, we highlight three unique aspects of a clinical case: The initial use of an endoscopic approach, followed by recurrence in the mastoid that necessitated mastoidectomy; the application of fused PET/MRI for monitoring and precise localization of residual disease; and the first documented presentation of cochlear–vestibular dysfunction in a MeNET case.

## Case Report

A 40-year-old male presented to a tertiary referral center with a left middle ear mass, profound sensorineural hearing loss (SNHL), and left vestibular hypofunction. The patient reported recurrent left ear infections with otalgia and temporomandibular joint pain, beginning 5 to 6 years prior to his initial visit. Additionally, he had an episode of left facial paralysis 4 to 5 years prior to presentation, which resolved with steroid therapy. On examination, the patient was found to have pulsatile tinnitus and persistent imbalance. Physical examination revealed a firm, tender flesh-colored mass in the left ear. The right ear canal was patent with a normal tympanic membrane. Facial function was intact and symmetrical. Left vestibular hypofunction was identified on clinical bedside examination; formal quantitative vestibular testing (e.g., video head impulse test (HIT), caloric testing, or videonystagmography) was not performed.

**Fig. 1:**
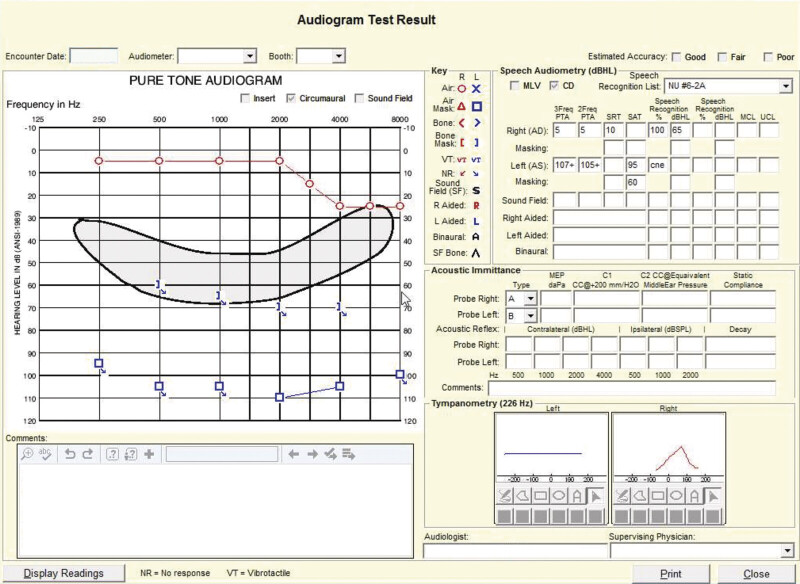
Audiogram demonstrating profound sensorineural hearing loss in the left ear.

**Fig. 2:**
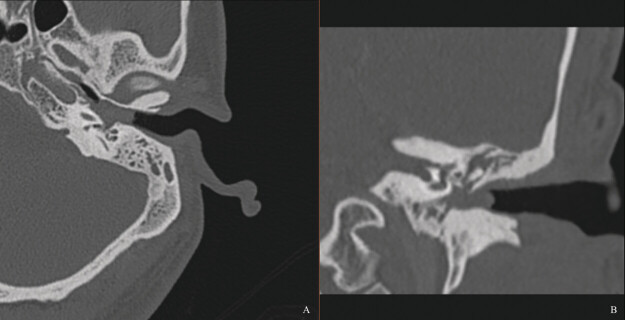
CT of the left temporal bone showing middle ear opacification and complete mastoid opacification with new bone formation around the oval window. ( **A** ) Axial view. ( **B** ) Coronal view.

**Fig. 3:**
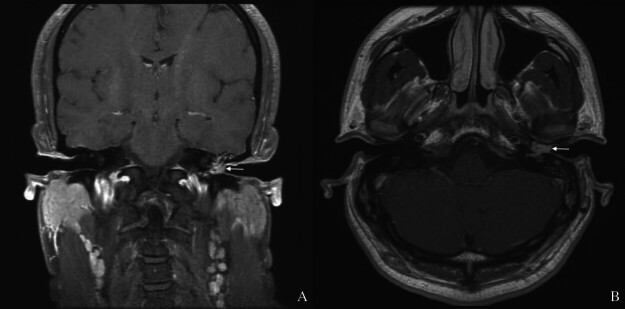
Magnetic resonance imaging showing left middle ear and canal enhancement. ( **A** ) Coronal view. ( **B** ) Transverse view. Mass (white arrows).

Audiometry confirmed profound SNHL in the left ear ( Fig. 1 ). Preoperative CT demonstrated a soft tissue mass filling the middle ear cavity and mastoid air cells, with new bone formation around the oval window ( Fig. 2 ). The lesion extended into the medial bony ear canal. The ossicular chain was intact, though the stapes appeared hyperostotic. The right temporal bone was normal. MRI further revealed a gadolinium-enhancing mass in the left middle ear and external auditory canal ( Fig. 3 ).

A biopsy of the ear canal lesion was performed in the clinic. Tumor cells were positive for chromogranin, synaptophysin, INSM1, and Cam5.2. They were weakly positive for AE1/AE3 (pan-cytokeratin monoclonal antibody) and negative for p40, SOX10, and S100. A K _i_ -67 proliferative index was estimated at 2%. Per the International Agency for Research on Cancer (IARC) and the World Health Organization (WHO) classification framework for neuroendocrine neoplasms, 3 the tumor was classified as a MeNET grade II.

The patient was taken to the operating room for a left endoscopic middle ear exploration with tympanoplasty and canalplasty for excision of the lesion. The tumor extended through the superior aspect of the tympanic membrane and filled the medial osseous ear canal. The tumor occupied the entire middle ear space and was densely adherent to the supratubal recess, oval window, round window, sinus tympani, sinus subtympanicum, subcochlear tunnel, and into a hypotympanic air cell track lateral to a visible pulsation that was identified as the petrous segment of the internal carotid artery. The ossicles were encased in a tumor, the malleus and incus were removed, and the stapes was fixed. A diode laser was used to remove the disease surrounding the stapes.

**Fig. 4:**
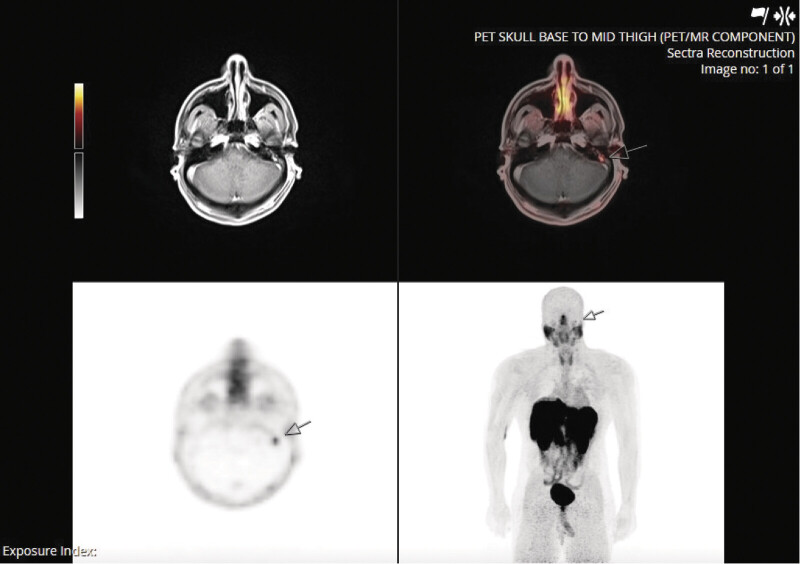
MRI/PET scan. Localized DOTA-TATE avidity (ma SUV 3.5) seen in the left mastoid (arrows) that was suspicious for residual DOTA-TATE avid neoplasm. ma SUV; maximum standardized uptake value.

Postoperatively, the patient's recovery was uncomplicated, though he was referred to vestibular therapy for persistent imbalance. A follow-up DOTA-TATE PET/MRI at 3 months showed evidence of persistent disease in the left mastoid ( Fig. 4 ). Given these findings, the patient was taken to the operating room for a mastoidectomy. The residual disease appeared to involve the mastoid antrum, facial recess, and hypotympanum, extending posteriorly to the sinodural angle. This was removed, and the facial recess was opened. A PET/CT scan 7 months postoperatively showed no residual or recurrent neuroendocrine tumor. Repeat bedside vestibular assessment following both the initial and revision surgeries revealed no catch-up saccades on head thrust, suggesting either persistent vestibular hypofunction or a compensated state rather than restoration of vestibular function. Dix–Hallpike testing postoperatively elicited a few beats of upbeat geotropic nystagmus without a torsional component on the right. Following a course of vestibular rehabilitation, an 8-point improvement on the Dizziness Handicap Inventory was recorded, consistent with the patient's subjective report of slight improvement in dizziness. A hearing aid evaluation was recommended to discuss potential contralateral routing of signals options for his left profound SNHL.

## Discussion

The presented case highlights the complexities of diagnosing and managing a MeNET, including atypical clinical presentations, the role of advanced imaging, and the potential need for second-look procedures. MeNETs are rare, and the most common symptoms are conductive hearing loss, ear fullness, tinnitus, and/or ear pain. 4 Facial nerve palsy has also been reported in some cases. 5 However, to our knowledge, cochlear–vestibular dysfunction has never been documented as a presenting symptom of MeNET. This case report represents the first reported instance of MeNET-associated cochlear–vestibular dysfunction.

The differential diagnosis for MeNETs is extensive and includes conditions such as jugulotympanic paraganglioma, schwannoma, and meningioma. 1 Therefore, a comprehensive workup, which consists of clinical evaluation, radiologic imaging, and ultimately histopathologic analysis with immunohistochemical staining, is necessary for diagnosis. In this case, histopathological findings from an in-office biopsy confirmed the presence of MeNET.

An endoscopic approach was initially chosen due to its advantages, including reduced morbidity, faster recovery, and superior visualization of key anatomical areas, such as the anterior epitympanum, sinus tympani, and facial recess. 6 In this case, the tumor was fully resected during the initial procedure, with no evidence of extension into the mastoid at that time. However, surveillance imaging with DOTA-TATE PET/MRI revealed residual disease activity in the mastoid antrum.

This pattern of residual disease aligns with existing literature, which describes a high recurrence rate in MeNETs despite seemingly complete resection. 7 8 9 Van der Lans et al reported recurrence in five out of eight patients following initial surgery, demonstrating the challenge of achieving complete clearance. 7 Given the unpredictable nature, careful postoperative surveillance and consideration of adjunctive therapies are essential. Nonetheless, definitive surgical resection remains the treatment of choice for MeNETs. Adjuvants such as somatostatin analogs (SSAs) have been explored in the setting of recurrent or metastatic MeNET. 10 11 Long-acting SSAs are well-established in the management of gastroenteropancreatic neuroendocrine tumors (GEP-NETs), where they exert both antisecretory and antiproliferative effects. 11 In 2021, the first successful treatment with long-acting SSAs was reported in two patients with postsurgical middle ear adenoma with neuroendocrine features that had recurred with metastases. SSA therapy was associated with a progression-free interval of approximately 1 year, comparable to outcomes observed with SSAs in GEP-NETs. 11 The same patients also received peptide receptor radionuclide therapy (PRRT), which achieved disease control for 15 months and at least 26 months. Another 2021 study similarly described two patients treated with ^177^ Lu-DOTATATE, reporting progression-free intervals of approximately 4 years after completing four cycles of therapy. 5 These reports collectively suggest that the systemic treatment framework established for GEP-NETs may be applicable in cases of unresectable or metastatic MeNET, though further prospective study is needed.

The patient's initial presentation with vestibular hypofunction and profound SNHL is also unique, especially given that the tumor had no evidence of inner ear involvement on either preoperative imaging or during surgery. Despite tumor resection, the patient experienced persistent imbalance postoperatively, requiring vestibular rehabilitation. 5 It should be noted that vestibular hypofunction in this case was identified on clinical bedside examination, without formal quantitative assessment. While bedside vestibular examination is a recognized and widely used clinical approach, the absence of quantitative data represents a limitation of this report. Vestibular function was also not formally reassessed following surgical intervention.

Several mechanisms may explain the cochlear–vestibular dysfunction observed in this case. One proposed hypothesis is that tumor-related disruption or involvement of the inferior cochlear vein is implicated in the development of SNHL. 12 This mechanism was originally described in jugular foramen paragangliomas, where pathologic involvement of the inferior cochlear vein was shown to correlate strongly with SNHL even in the absence of otic capsule invasion. 12 The inferior cochlear vein is the main outflow tract from the cochlea and part of the vestibular labyrinth. Although clinical reports delineating inferior cochlear vein disruption in the context of vestibular dysfunction are limited, other case reports and series have described acute vestibular symptoms following venous outflow obstruction, such as in cerebral venous thrombosis. 13 14 In our case, no direct labyrinthine invasion was noted on imaging, but the tumor's encasement of structures near the round window could theoretically place the adjacent inferior cochlear vein at risk.

A second possibility is that the cochlear–vestibular dysfunction is from secondary labyrinthitis due to microscopic invasion of the otic capsule. While such behavior has not yet been reported in MeNETs, similar inner ear involvement has been documented in other middle ear tumors, such as paragangliomas and cholesteatomas. 12 15 In our case, persistent vestibular hypofunction despite tumor resection raises the possibility that a subclinical inflammatory process may have extended into the membranous labyrinth.

The limited literature on MeNETs hinders the development of standardized diagnostic and management strategies. This report contributes the first documentation of MeNET-associated cochlear–vestibular dysfunction and highlights the value of fused DOTA-TATE PET/MRI imaging in detecting residual disease. Continued case reporting is essential to improve understanding and management of this rare tumor.
